# How bioinformatics influences health informatics: usage of biomolecular sequences, expression profiles and automated microscopic image analyses for clinical needs and public health

**DOI:** 10.1186/2047-2501-1-2

**Published:** 2013-01-10

**Authors:** Vladimir Kuznetsov, Hwee Kuan Lee, Sebastian Maurer-Stroh, Maria Judit Molnár, Sandor Pongor, Birgit Eisenhaber, Frank Eisenhaber

**Affiliations:** 1 Bioinformatics Institute (BII), Agency for Science, Technology and Research (A*STAR), 30 Biopolis Street, #07-01, Matrix, 138671 Singapore; 2 School of Computer Engineering (SCE), Nanyang Technological University (NTU), 50 Nanyang Drive, Singapore, 637553 Singapore; 3 School of Biological Sciences (SBS), Nanyang Technological University (NTU), 60 Nanyang Drive, Singapore, 637551 Singapore; 4 Institute of Genomic Medicine and Rare Disorders, Tömö Street 25-29, 1083 Budapest, Hungary; 5 Faculty of Information Technology, Pázmány Péter Catholic University, Budapest, Hungary (PPKE), Práter u. 50/a, 1083, Budapest, Hungary; 6 Department of Biological Sciences (DBS), National University of Singapore (NUS), 8 Medical Drive, Singapore, 117597 Singapore

**Keywords:** Genome sequencing, Expression profiling, Histopathological bioimaging, Bioinformatics, Cancer mutation, Cancer biomarker, AIDS, HIV, Influenza, H1N1, Enterohemorrhagic *Escherichia coli*, Quorum sensing, Digital pathology, Glaucoma, Dry eye, Tumor segmentation

## Abstract

**Abstract:**

The currently hyped expectation of personalized medicine is often associated with just achieving the information technology led integration of biomolecular sequencing, expression and histopathological bioimaging data with clinical records at the individual patients’ level as if the significant biomedical conclusions would be its more or less mandatory result. It remains a sad fact that many, if not most biomolecular mechanisms that translate the human genomic information into phenotypes are not known and, thus, most of the molecular and cellular data cannot be interpreted in terms of biomedically relevant conclusions. Whereas the historical trend will certainly be into the general direction of personalized diagnostics and cures, the temperate view suggests that biomedical applications that rely either on the comparison of biomolecular sequences and/or on the already known biomolecular mechanisms have much greater chances to enter clinical practice soon. In addition to considering the general trends, we exemplarily review advances in the area of cancer biomarker discovery, in the clinically relevant characterization of patient-specific viral and bacterial pathogens (with emphasis on drug selection for influenza and enterohemorrhagic *E. coli*) as well as progress in the automated assessment of histopathological images. As molecular and cellular data analysis will become instrumental for achieving desirable clinical outcomes, the role of bioinformatics and computational biology approaches will dramatically grow.

**Author summary:**

With DNA sequencing and computers becoming increasingly cheap and accessible to the layman, the idea of integrating biomolecular and clinical patient data seems to become a realistic, short-term option that will lead to patient-specific diagnostics and treatment design for many diseases such as cancer, metabolic disorders, inherited conditions, etc. These hyped expectations will fail since many, if not most biomolecular mechanisms that translate the human genomic information into phenotypes are not known yet and, thus, most of the molecular and cellular data collected will not lead to biomedically relevant conclusions. At the same time, less spectacular biomedical applications based on biomolecular sequence comparison and/or known biomolecular mechanisms have the potential to unfold enormous potential for healthcare and public health. Since the analysis of heterogeneous biomolecular data in context with clinical data will be increasingly critical, the role of bioinformatics and computational biology will grow correspondingly in this process.

## When will genome sequences, expression profiles and computer vision for bioimage interpretation be routinely used in clinical medicine?

There is apparently no doubt for anyone that modern life science research based on the new high-throughput technologies most prominently represented by genomic sequencing together with the increasingly powerful and, at the same time, affordable information technology products will dramatically change healthcare. The main idea behind these expectations is that the new availability of data characterizing the patients’ individuality at the level of genome, biomolecules and gene/protein networks together with evermore powerful diagnostic, mainly imaging tools at the histological, anatomical and physiological levels allow ever finer stratification of the patients’ conditions once the molecular data is integrated with clinical data and, finally, it will lead to the design of personalized treatment regimes.

Unfortunately, the discussion in the media has become hyped with expectations increasingly getting out of touch with the progress that both biomedical science [1] and healthcare at the ground can deliver in the short and medium term. In this discussion and, to some extent, review article, we try to analyze what are major trends in computational biology and bioinformatics that support the advance towards stratified and personalized medicine and what are the fundamental and some of the procedural barriers on the path towards the solution of major healthcare problems such as infections, cancer, metabolic and neurodegenerative diseases, familial disorders, etc.

The article is structured as follows: In the section The hype around genomics and proteomics technologies in the healthcare context and fundamental reasons calling for a temperate view, we look into the general developments that fuel the expectations of revolutionary change in health care and public health; we talk about several roadblocks that have been removed on the path towards personalized/stratified medicine and the possible role of bioinformatics and computational biology in this process. We also emphasize what are the reasons why many of the expectations will not materialize in the short- to medium-term time frame. Section Management of innovation cycles of high-throughput technologies and the role of bioinformatics in this process is dedicated to issues that arise when bioinformaticians/computational biologist actually penetrate into the actual health care provision system under the condition when the application of new computational analysis methods and evaluation protocols is not really routine.

In sections Bioinformatics moving towards clinical oncology: biomarkers for cancer classification, early diagnostics, prognosis and personalized therapy (cancer biomarkers), Sequence-structure-function relationships for pathogenic viruses and bacteria and their role in combating infections (infectious diseases) and Impact of Bioimage Informatics on Healthcare (computerized histopathology), we exemplarily discuss and partially review the progress in application areas that have already or will likely benefit in the near future from interaction with bioinformatics/computational biology approaches. Although often histologically similar, increasingly more cancer subtypes are getting characterized at the level of the specific, individual biomolecular mechanisms that drive the growth of the tumor cell population and, thus, are essentially understood as different diseases. Cancer biomarkers are critical for diagnosis, classification, prognosis and therapy progress evaluation in this concept (section Bioinformatics moving towards clinical oncology: biomarkers for cancer classification, early diagnostics, prognosis and personalized therapy).

Due to their small genome and the possibility to successfully deduce phenotype properties from mutations, viral and bacterial pathogens are thankful objects for computational biology analysis in the clinical context (in contrast to the situation with higher eukaryotes such as human; section Sequence-structure-function relationships for pathogenic viruses and bacteria and their role in combating infections). As example, we review in depth the clinically relevant characterization of patient-specific influenza viral infections. We also show that genome analysis of enterohemorrhagic *E.coli* allows selecting existing FDA approved drugs for treatment.

In section Impact of Bioimage Informatics on Healthcare, we review advances in the automated assessment of histopathological and, to a minor extent, other medical images. Possibly, these developments in this area might have a non-spectacular but a very profound impact on health care delivery very soon since the problems to overcome are more of the engineering type and not of fundamentally scientific origin.

### The hype around genomics and proteomics technologies in the healthcare context and fundamental reasons calling for a temperate view

Several roadblocks towards the goal of stratified/personalized medicine have disappeared very recently. The spectacular improvement of nucleic acid sequencing technologies lead to a reduction in costs, both in time and money, at a scale that can only be described as jaw-dropping for the observer. Whereas the first full human genome sequencing absorbed about 3 billion USD in the USA alone and it took about a decade to be accomplished [2], recently offered machines such as Ion Proton™ Sequencer (Life Technology) or HiSeq™ 2500 (Illumina) [3] move these numbers rather close towards 1000 USD and a single day. And this appears not to be the endpoint of the technology development with more progress to be expected in the medium-term future. Naturally, dreams about all kinds of sequencing applications, especially, in clinical contexts and with affluent patients start sprouting. To note, the progress of nucleic acid sequencing is just the most eye-catching; essentially, it hides dramatic progress also in many other areas and high-throughput technologies such as expression profiling, histopathological image processing, etc. We need to acknowledge, that for life sciences, where, historically, getting at least some verifiable, quantified data for their biological system of study was a major difficulty and the setup of experiments and not the analysis of the measurement absorbed most of the intellectual capacity [4], the current deluge of quantified data is really a game changer and puts theoretical analysis detached from experimentation into general importance for the field for the first time.

The second major change is in IT itself. The older among the list of authors still remember their times as PhD students when the access to mainframe machines was cumbersome and heavily restricted and a good desktop computer with graphical interface in the late eighties/early nineties had the price of a luxury sports car. Today, for nominally the same money, one can equip several research teams if not a small institute with computer clusters (e.g., a 64 core computer trades for just about 10000 USD), storage systems and network tools that are more powerful than necessary for about 90% of the tasks in computational biology. Thus, computing and storage opportunities are essentially no longer the limiting factor for life science research compared with just a decade or even a few years ago.

The hype currently accumulating around the new opportunities with sequencing and other high-throughput technologies, maybe, is sensed most directly in the entrepreneurs’ and scientists’ comments compiled by Bio-IT World at its WWW page dedicated to the 10^th^ anniversary of its own launch [5]. Although there are some minority cautionary notes, one cannot get away with the general impression that concluding from molecular data to clinically important statements is mainly seen as a problem of the scale of data generation. It is expected that the IT-centric efforts of integrating patient-specific sequencing, expression, tissue imaging data with clinical information (whatever might be the exact meaning of this “data integration”; just putting everything into one electronic database) will inevitably lead to significant healthcare outcomes in terms of personalized medicine.

This surprisingly optimistic view remembers the euphoria that, ten years ago, accompanied the presentation of the first draft of the human genome caused by the anticipation that “Genetic prediction of individual risks of disease and responsiveness to drugs will reach the medical mainstream in the next decade or so. The development of designer drugs, based on a genomic approach to targeting molecular pathways that are disrupted in disease, will follow soon after” [6]. With hindsight, we know that the progress in the last decade has not reached the promises, not even nearly [1, 7]. The hype in the media is also in suspicious contrast to the recent attempt of certain pharmaceutical companies to slash down their own research force and to promote the idea of open innovation, i.e., essentially unloading research efforts, costs and research risks into the public sphere.

Whereas the general developmental trend appears correctly predicted, the devil is in the detail and the serious disagreement is about timescales and in which areas/applications the healthcare breakthroughs from genomics and other technologies are more likely in the time closer to us. Moving from the scientific laboratory to actual healthcare is also associated with a myriad of additional issues besides the scientific task itself. Apparently boring questions such as predictive power, robustness, standardization, availability and reliability of the new methods in conditions of routine application in regular hospitals, clinics and in the out-patient context by possibly scientifically insufficiently trained personnel become urgent. This includes the comparison of the new methods with more traditional, tested approaches not only from the viewpoint of medical science but also cost-wise (in terms of money and working time for tests and data analyses). Since considerable economic interest is associated with the upcoming healthcare revolution not only from IT equipment and healthcare solution providers but also from charlatans who, for example, try to sell life style advice derived from the customers’ own genome sequence already today, it is important to get the discussion away from the level of fairy tale and hyped promises and to assess the current state of the art realistically.

Besides the costs, the most important argument against having genome sequencing and expression profiling from every patient at present is the fact that the overwhelming part of this data cannot be interpreted into biologically and/or medically significant conclusions. Today, ever faster sequencing leads foremost to ever faster growing amounts of non-understood sequence data. To note, we need to know about the biomolecular mechanisms that translate the genome sequence into phenotypes when we wish to interfere rationally at the molecular level. As elaborated elsewhere, the biological functions of about every second human gene are not well or even completely not known [1]. The whole mystery of non-coding RNA function is hardly scratched upon; yet, we know that many, also non-protein-coding regions of the genome are actively transcribed and this expression influences important biological processes [8, 9]. Maybe, it was one of the most important insights from the whole human genome sequencing project that we can estimate now how much human biology at the molecular level we do not know, namely most likely (much) more than 50% [1]. To just search for correlations between phenotypic, including clinical conditions and genomic changes will appear insufficient because of several reasons: 1) the path relating genome features and phenotype is extremely complex in many cases. 2) The statistical significance criteria will require impossibly large cohorts. 3) Rationally designed therapy without mechanistic insight is problematic. Given the pace of progress in the area of biomolecular mechanism discovery during the last decade, it is expected that it will take another century until we will understand our own genome. Presumably, scientific, technological and social factors will kick in that will accelerate the advance [1]; yet, it is clear that this is not a short term issue.

Most likely, biomedical applications that rely either on the comparison of DNA or, generally, nucleic acid sequences, without necessarily understanding their biological meaning or on the biomolecular mechanisms that are already more or less known have the greatest likelihood to achieve importance for healthcare, public health and biotechnology. To the first class of applications belong methods for the identification of the human individual’s origin and identity, be it in the forensic, genealogy or legal context, but also the diagnostics of hereditary diseases and the characterization of food items in terms of quality and origin. With regard to the latter class of applications, those diseases that require the investigation of less complex gene networks and biomolecular mechanisms will have better chances to benefit from sequencing, expression profiling and histopathological imaging informatics than those with more complex mechanisms. In this light, the perspectives of fighting infections or cancer are more promising than, for example, those of battling obesity since energy metabolism appears to be one of the most complexly regulated systems in humans.

In this context, does the sequencing of patients’ DNA in a large scale make sense? In several countries, for example in Norway [10], programs are being implemented that aim exactly at realizing this vision, the sequencing of the patients’ genomes and of their cancers. It appears to us that, at this stage, the move may be justified for small, rich countries that have the necessary capacity to finance an extensive follow-up fundamental research effort to study the newly collected data since, in many cases, the clinical outcome for the specific patient might be negligible at present. Thus, sequencing, expression profiling, etc. make sense in a clinical setup where the data can enter into a research environment for proper, non-standard data analysis and where, beyond potential benefit for the specific patient, these expensive laboratory investigations can have serendipitous consequences for the scientific knowledge gain that might benefit many other future patients.

### Management of innovation cycles of high-throughput technologies and the role of bioinformatics in this process

In addition to fundamental scientific problems with biomolecular mechanisms discovery, we need to emphasize that high-throughput technologies such as nucleic acid sequencing are far from mature. The renewal cycle involves maximally a couple of years and it might be already tomorrow that, due to some unexpected innovation, the equipment purchased yesterday is hopelessly out of date even if the machines continue to look shiny. Since the new generation of sequencing, expression profiling and other high-throughput technologies tend to generate the biological data at much lower costs and with higher accuracy than their predecessors, it does not make sense to produce more data than can be properly analyzed within a reasonably short time frame; future researcher will rather look at regenerated data produced with newer technologies available then instead of reviving old data files.

Even for dedicated research institutions with rich budgets, it remains a financial problem to participate in every step of technology development. It is not just the purchase of new pieces of equipment, but also the establishment of subsequent data analysis pipelines, software replacements and the training of the respective staff or even the hiring of new types of professionals. The latter issues might create more headache than the sequencer purchase itself.

Many clinical labs attached to research and other top-end hospitals around the world are thinking about how to prepare for a swift increase in genomics and proteomics analysis needs. Ever since their emergence in 2005, next-generation sequencing (NGS) technologies have proven revolutionary research tools in a variety of scientific disciplines of the life sciences. NGS technologies are now increasingly being applied in clinical environment, which is partly due to the emergence of novel and efficient sequencing protocols and partly to the appearance of smaller, less expensive sequencing platforms. The possibilities of applying NGS in clinical research ranges from full human genome profiling [11], microbiome profiling [12] to biomarker discovery, stratification of patients for clinical trials, prediction of drug response and patient diagnosis. Such applications often involve targeted re-sequencing of genes of clinical relevance whereby not the entire genome is sequenced, only a few dozen PCR-amplified regions or known disease-related genes. These genes harbor diagnostic or causative mutations of diseases including indels and single nucleotide polymorphisms. Individual genes have previously been interrogated in clinical testing using traditional techniques such as Sanger sequencing however NGS technologies have already begun to supplant the previous tools of choice in these areas, offering increased speed and throughput with reduced running costs.

Targeted re-sequencing in the clinical context presents specific requirements and new challenges also for bioinformatics which is aggravated by new computational needs of fast changing sequencing platforms. Just to mention one problem, that of multiplexing: simultaneous analyses of many patients for many diseases require accurate and unequivocal identification of many persons and many genes within an ensemble of many hundred thousand reads. Molecular bar-coding makes this possible, but standard bioinformatics tools are not ready to handle bar-coding information [13, 14].

Clinical labs seek the advice of bioinformaticians regarding what kind of software to use. The usual standard answer is to use the current best of genomics software. Unfortunately, it is often found that these tools are not even always capable of doing the clinical application job, for example detecting specific mutation types. The reason is simple: Genome aligners were designed to map short reads to a whole genome, i.e., finding relatively strong similarities in a background of weak or minimal similarities. This scenario has called for specific speed-up solutions and approximations, many of which may not necessarily be true for amplicon sequencing protocols. So, clinicians usually face two problems: i) Buy an expensive hardware and non-transparent, and more often than not, very computer time-consuming commercial software from the platform vendor, or ii) seek advice from trained bioinformaticians who may point them to academic tools developed for genome analysis, but not necessarily suitable for amplicon sequencing. The solution is not easy. Platform vendors cannot be blamed for proposing a technically sound solution which, for the moment, has no chances to follow the exponential growth of clinical analysis needs. So, it is the task of future bioinformatics projects to develop accurate and flexible solutions for clinical applications.

## Bioinformatics moving towards clinical oncology: biomarkers for cancer classification, early diagnostics, prognosis and personalized therapy

Losses of human lives and sufferings as a result of cancer remain one of the critical obstacles in prolonging active human life span. Worldwide, cancers are responsible for one in eight deaths [15]. In Singapore, cancers are the major causes of mortality and accounts for about 28.5% of all deaths [16]. In our present understanding, cancer is a disease involving genetic changes in certain cell populations that lead to cellular reprogramming and uncontrolled cell division; in turn, the formation of a malignant mass can create a variety of clinical symptoms. The huge individual genome variation and diversity of cellular phenotypes in cancers often complicates clinical detection, classification, prognosis and treatment of patients. In fact, histologically similar cancers do not necessarily represent the same disease due to differences in the biomolecular mechanisms leading finally to similar clinical outcomes. Consequently, among the list of 10 most important human diseases, the pharmacotherapy efficacy of cancer is very low except for a few rare subtypes [17]. The progress in the early diagnostics/detection and therapy of many cancers is very slow. For instance, for the past 30 years, ovarian cancers (OC) mortality rate has remained very high and unchanged, despite considerable efforts directed toward this disease.

Current clinical oncology needs (i) improvement of disease classification, (ii) increased specificity and sensitivity of early detection instruments/molecular diagnostics systems, (iii) improved disease risk profiling/prediction, (iv) improvement of cancer therapeutic methods including next generation drugs with higher specificity and lowered toxicity (ideally, inhibitors of the exact biomolecular mechanisms that drive individual cancer growth) and generally more stratified or even personalized therapies, (v) understanding of the anti-cancer immune response, (vi) adequate monitoring and rehabilitation during post-treatment recovery period and (viii) patients’ social adaptation.

At present, there are two main lines of support for clinical oncology from the side of computational biology fuelled by data generated by genomics and proteomics high-throughput technologies. On the one hand, genome and RNA sequencing as well as expression profiling of cancer biopsy samples opens the possibility to understand the biomolecular mechanisms that are behind the malignant transformation in the individual patient’s tumor case. On the other hand, the status of biomarkers can be measured and used to provide more accurate diagnostics of a specific cancer type, prognosis and selection of personalized therapy.

### Hunting after cancer mutations in a clinical setup

The problems associated with large-scale sequencing and expression profiling of cancers need to be seen from two sides. Whereas the technical aspects of correct sequence and expression profile determination from generally miniscule biopsy amounts are considerable but manageable (see a recent review of some of the IT and bioinformatics aspects [18]), the evaluation of the data in terms of clinically relevant conclusions for the specific patient is presently impossible in most cases and the clinically relevant effort is centered more around the question whether the actual patient happens to carry a cancer that belongs to one of the better understood subtypes. At the same time, sequencing and expression profiling of carefully selected cohorts of cancer patients are of immeasurable value for biomedical research aimed studying yet unknown biomolecular mechanisms.

Technically, analyzing somatic mutations in complex diseases such as cancer is particularly challenging since the mutant alleles can be easily diluted below detection thresholds due to the presence of wild type non-tumor DNA and the inherent genetic heterogeneity of the tumor itself. The problem is further aggravated by the limited amount of DNA (1-100 ng) available from biopsies on the one hand, and the clinical sample preparation, on the other: For example, clinical samples fixation in formalin randomly breaks DNA into 200-400 bp long fragments.

The current gold standard method tries to circumvent these problems by applying targeted PCR amplification to 100-200 bp long target sequences which is followed by Sanger sequencing of the PCR amplicons. Next generation sequencing (NGS) platforms such as the 454 FLX Genome analyzer (Roche) or Ion Torrent Personal Genome Machine (Life Technology), offer important advantages due to their extremely high (1000-10000 fold) sequence coverage. Thus, sensitivity as compared to Sanger sequencing is increased. This is very important for detecting low frequency mutations, which makes NGS an attractive option for diagnostic sequencing.

For clinical analysis of the transcriptome, deep sequencing technologies (e.g. RNA-seq, etc.) allow detecting low abundant RNA transcripts. Many classes of these transcripts (e.g., long non-coding RNAs) play essential regulatory roles in cancer development and can potentially be used for clinical sub-typing, detection, prognosis and therapy design of cancers. Detection of the rare genome aberrations and low-abundant transcripts in cancers and in human body fluids might be important. However, clinical studies of such data require development of appropriated biomedical research infrastructure, collection of large patients’ cohorts, management of well-coordinated interdisciplinary research projects, dynamical and integrative databases, novel IT solutions and massive data analyses within a computational biology research effort.

Another advantage of NGS technology is its ability to deal with parallel sequencing of multiple genes. The widely respected white paper of the American Society of Clinical Oncology [19] suggested that all targeted drugs should be registered based on the molecular profile independently from the tumor type. Recently, researchers of the Massachusetts General Hospital argued that simultaneous analysis of 12 genes is useful for the diagnosis of lung cancer [20]. Therefore, there is a clinical need for targeted re-sequencing of dozens of genes in each cancer patient. There are several, commercially available multiplex re-sequencing assays in clinical use today. A typical analysis for cancer targets may require PCR-based re-sequencing of 10 to 1500, mainly exon-derived amplicons selected from 10 to 400 genes, and a minimum amount of 10 ng DNA [21].

### Biomarkers for cancer classification: mutations in signaling proteins

A biomarker is a traceable biochemical substance that is informative about the status of a disease or medical condition. For practical purposes, it is sufficient to show a close correlation between the occurrence of the biomarker and the cancer type and development in model systems and in clinical trials. Yet, the likelihood of the biomarker actually being associated with the cancer subtype considered is dramatically increased if the biomarker plays a role in the biomolecular mechanisms driving the cancer and not just in some secondary or tertiary effects of cancer growth. However, discovery of reliable diagnostic, prognostic and drug response cancer biomarkers faces big challenges due to patient heterogeneity, small sample sizes, and high data noises.

A couple of cancer subtypes well-characterized mechanistically have recently seen spectacularly successful treatment. Mutations in signaling proteins have been found to drive cells into the cancer state and the design of drugs that specifically bind to these mutated forms have been shown to suppress cancer development. For the drugs to be applied, a companion diagnostic test is necessary to verify whether the potential patient has indeed a cancer driven by the target supposed. As a rule, this will dramatically shrink the number of patients but the selected ones have a high chance to receive benefits from the treatment. Three cases illuminating the trend towards mutation-specific targeting drugs are reviewed in some detail below.

Several forms of chronic myelogenous leukemia (CML) and gastrointestinal stromal tumors (GISTs) are characterized by the Philadelphia chromosome, a chromosomal translocation, and the subsequent fusion of genes bcr and abl. As a result, the tyrosine kinase abl is locked in its active signaling state and affecting the downstream pathways Ras/MapK (increased proliferation due to increased growth factor-independent cell growth), Src/Pax/Fak/Rac (increased cell motility and decreased adhesion), PI/PI3K/AKT/BCL-2 (suppression of apoptosis) and JAK/STAT (driving proliferation). The inhibitor Imatinib (STI571, Gleevec) inhibits bcr-abl and, as a result, an originally fatal disease is transformed into a chronically manageable one [22]. The same inhibitor is also active for some sequence variants of c-kit and PDGF-R (platelet-derived growth factor receptor) and, thus, can be applied in a handful of other cancers. Since application of the drug is essentially selectively killing sensitive cells, strains with resistant mutations survive and it might require the application of other batteries of drugs to bring these strains down, too [23].

Another case with some success are melanoma subtypes with the B-RAF mutation V600E that can be treated with vemurafenib (PLX4032, RG7204) [24, 25]. In melanomas with mutant B-RAF (V600E), the drug inhibits specifically B-RAF (V600E) monomers. Since the ERK signaling inhibition is tumor-specific, these RAF inhibitors have a broad therapeutic index and a remarkable clinical activity in patients with melanomas that harbor the respective B-RAF mutant (V600E). However, resistance invariably emerges, for example via alternative splicing. The version p61 B-RAF (V600E) shortened by exons 4-8 shows enhanced dimerization in cells with low levels of RAS activation and ERK signalling is resistant to the RAF inhibitor [25].

Certain EGFR (epidermal growth factor receptor, another tyrosine kinase) driven cancers of breast, lung, pancreas, etc. are sensitive to gefitinib (Iressa) or erlotinib (Tarceva). The EGFR class includes Her1 (erb-B1), Her2 (erb-B2), and Her 3 (erb-B3). The EGFRs are hyper-activated due to a mutation in the tyrosine kinase domain and this leads to inappropriate activation of the anti-apoptotic Ras signalling cascade, eventually resulting in uncontrolled cell proliferation [26].

### Biomarkers for cancer classification: up-regulated genes

The literature on cancer biomarkers is enormous and it is beyond this review to be comprehensive. Here, we focus on developments with our authors’ involvement.

Lung adenocarcinoma (AC) is the most common type of lung cancer which is the leading cause of cancer deaths in the world. The genetic mechanisms of the early stages and lung AC progression steps are poorly understood. Currently, there are no clinically applicable gene tests for early diagnosis and lung AC aggressiveness assessment. Recently, authors of this review (VK *et al*.) suggested a method for gene expression profiling of primary tumours and adjacent tissues (PT-AT) based on a new rational statistical and bioinformatics strategy of biomarker prediction and validation, which could provide significant progress in the identification of clinical biomarkers of lung AC. This approach is based on the extreme class discrimination (ECD) feature selection method that identifies a combination/subset of the most discriminative variables (e.g. expressed genes) [27]. This method includes a paired cross-normalization (CN) step followed by a modified sign Wilcoxon test with multivariate adjustment carried out for each variable. Analysis of paired Affymetrix U133A microarray data from 27 AC patients revealed that 2,300 genes can discriminate AC from normal lung tissue with 100% accuracy. Our finding reveals a global reprogramming of the transcriptome in human lung AC tissue versus normal lung tissue and for the first time estimates a dimensionality of space of potential lung AC biomarkers. Cluster analysis applied to these genes identified four distinct gene groups. The genes related to mutagenesis, specific lung cancers, early stage of AC development, tumour aggressiveness and metabolic pathway alterations and adaptations of cancer cells are strongly enriched in the discriminative gene set. 26 predicted AC diagnostic biomarkers (including SPP1 and CENPA genes) were successfully validated on qRT-PCR tissue array. The ECD method was systematically compared to several alternative methods and proved to be of better performance [27]. Our findings demonstrate that the space of potential clinical biomarker of lung cancers is large; many dozens of combined biomarkers/molecular signatures are possible. This finding suggests that further improvement of computational prediction and feature selection methods is necessary in conjunction with systematic integration of massive and complex data analysis.

Similar computational approaches applied on breast cancer patients’ expression data allowed important new insights into molecular and clinical classification, tumor aggressiveness grading and identification of novel tumor sub-types. Current statistical approaches for biomarker selection and signature extraction were extended by developing a hybrid univariate/multivariate approach, combining rigorous statistical modeling and network analysis [28]. In this approach, single survival-significant genes can be identified and used to generate important cancer related gene networks. The method also allows estimating the synergistic effect of two or several genes belonging to the same or different networks on the patients’ survival. With this analysis, we generated and evaluated several related signature sets which are superior to traditional clinical prognostic markers and existing breast cancer classifications [28–30]. The final groupings have significantly different p53 mutation status, tumor aggressiveness grading and metastasis events. Most importantly, it could be shown that the intermediate class of G2 breast cancers does not have a justification at the level of gene expression. The G2 cases are shown to be either G1-like or G3-like. This implies that G2 patients with a G3-like expression profile are recommended to receive the more aggressive treatment reserved for G3 patients.

Currently, using clinical and molecular markers does not provide specific and reliable ovarian cancer (OC) patients’ stratification, prognosis and treatment response prediction. High-grade epithelial ovarian serous carcinoma (HG-EOC), a major type of OC, is poorly detected. At the molecular level, the tumors frequently exhibit altered expressions of many hundreds and thousands features at genome, transcriptome and proteome levels. The specific and reliable biomarkers of this complex disease and appropriate therapeutic targets have not been defined yet. Similar computational approaches as described above in the cases of lung and breast cancers have been used to derive expression signatures for OC and they were found to include the EVI1 gene [31].

It is also notable that non-coding RNAs can also be used as biomarkers [32]. To conclude, the identification of reliable diagnostic, prognostic and drug response-related biomarkers for cancer requires integrative data analysis and understanding of the molecular and cellular basis of genome loci and gene expression and pathways.

## Sequence-structure-function relationships for pathogenic viruses and bacteria and their role in combating infections

Whereas the discussion above has highlighted that sequence-function relationships are not well understood and this status will continue for a while, the situation for the small genomes of pathogenic viruses and bacteria is considerably more promising. Their genome size is much smaller (from a handful of genes in the case of viruses to maximum a few thousand genes for bacteria) and their physiology is much more completely understood at the level of biomolecular mechanisms. For example, there is no gene in the influenza virus where at least some mechanistic aspect of its molecular and cellular function is known; a stark contrast to the situation for the human genome where about half of the genes still await their at least initial characterization [1] and even the compilation of the complete proteome is not in sight [33].

With sequencing getting increasingly cheaper and efficient, it became possible to explore the full genome of the set of strains that is actually invading the patient’s body. This is important since, to evade the patient’s immune system, the pathogen mutates and one or several of the mutants might find the weak spots of the patient and propagate. This allows not only designing efficient patient-specific treatment strategies, for example by deducing certain drug resistances theoretically from the pathogen’s genomic sequence before even trying actually the respective drug in the treatment. It provides also much better options for epidemiology and public health since each strain can be individually determined and, thus, the actual spread of the pathogen can be traced geographically and in real time. Measures for preventing and combating epidemics can be designed more rationally and with lower costs for social and economic life.

Most attention with regard to rationally designed strategies for fighting infection so far has been directed towards the acquired immunodeficiency syndrome (AIDS) caused by the human immunodeficiency virus (HIV) and this can rightly be considered a success story for computational biology. A previously absolutely fatal disease has been transformed into a chronic illness with high quality of life and, for many patients, with apparently zero viral blood counts. Not only have all the drugs against AIDS used in the multi-drug cocktail for high active antiretroviral therapy (HAART) been rationally designed against structures of HIV proteins to interfere into the well-studied life cycle of the virus [34]. New drugs appear all the time and provide new treatment opportunities for patients harboring strains resistant against the standard cocktails [35]. Sophisticated knowledge-based therapeutic algorithms [36] are available to treat AIDS patients optimally depending on the mutation spectrum within the patient’s viral load [37, 38].

Similar strategies are useful for other pathogens that try to evolve away from the attack of antibiotics/antiviral therapy or the immune system’s efforts. *Staphylococcus aureus* causing a wide range of infection from skin to post-operative wound infections has great adaptive potential and can generate forms (best known as methicillin-resistant *Staphylococcus aureus* - MRSA) widely resistant against many available antibiotics. Exact determination of the molecular epidemiology with multi-locus sequence typing and other methods can be the basis for an optimized antibiotics selection for more efficient therapy [39].

In the following, we explore how classical bioinformatics aimed at studying biomolecular sequences and structures can impact infection medicine in context with the influenza virus and the enterohemorrhagic *E. coli* pathogens.

### Genome sequence studies of the influenza virus and public health

Besides the occasional pandemics, recurrent seasonal influenza and its ongoing evolution has always been an important topic concerning public health. Whenever a new flu strain emerges and threatens to circle the globe, health authorities and clinicians need to know the characteristics of the new virus including virulence, drug susceptibility and vaccine efficacy. The recent swine flu pandemic from 2009 is an excellent example how computational methods can provide crucial support not only in the early molecular characterization [40–42] but also to follow the still ongoing evolution of the virus. Modern sequencing technology and increased preparedness resulted in a significant worldwide increase of institutions and hospitals that can generate molecular sequence data from patient samples. But when the patient-specific strain sequences are available after sequencing ordered by hospitals or ministries, it appears that the institution cannot properly handle them. The expertise for the subsequent steps of computational analysis to connect the genotype to possible phenotypes is often sparse. Bioinformatics can be used to rapidly screen influenza sequences for potentially interesting mutations, for example, through comparative genomics, 3D structural modeling, literature text mining and plotting geo-temporal occurrence patterns for epidemiological significance.

While this sounds exciting, are we really in a state that we can reliably predict relevant phenotypic changes from sequence mutations? First, the influenza genome is small and codes for only 10-13 proteins all of which are well characterized in their functions and there exists a mechanistic understanding how they work together as well as how they interact with the infected host. Second, there is wide interest in influenza research and the amount of available sequences, crystal structures, experimental data and associated literature is enormous which allows transferring information and annotations if very closely related strains are compared. For example, the typical Tamiflu resistance mutation H274Y in the neuraminidase protein has the same effect on equivalent positions in seasonal H3N2, old seasonal H1N1, pandemic H1N1, avian H5N1, etc.

**Figure 1:**
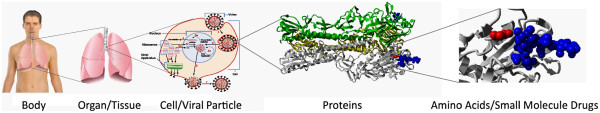
**The link between an influenza virus mutation and the altered course of infection.** Schematic representation showing how a single viral amino acid mutation (right, red balls) can affect host cell receptor (blue balls) interaction, which can alter viral localization and where the infection takes place, which in turn can affect severity and symptoms for the patient (left). A thorough understanding of the effects of mutations on biological mechanisms is also important for other human diseases such as cancer as well as patient-specific response to different treatments. Attribution of images: The 3 left-most images of the composed figure are public domain or under free-to-use licenses at Wikimedia commons from the following sources: patient body and organ [118] and infected cell [119].

But what can be said about “new” mutations? In the second wave of the 2009 H1N1 pandemic, a Norwegian team reported a high frequency of a new hemagglutinin mutation D222G in severe cases [43]. The power of bioinformatics for linking genotype to phenotype for influenza mutations can be shown for this example, as within a few hours from first reports of the mutation one could find a possible mechanistic explanation on how this mutation could possibly exert its severity using computational tools and databases alone. The first obstacle is the numbering, different groups prefer to use old seasonal H3N2 based numberings also for H1N1 pandemic strains but it is important to know that D222G is actually corresponding to the mutation D239G in the literal sequence numbering of circulating pandemic strains which is necessary to find and count appearances of this mutation in available influenza surveillance sequences. This can easily be resolved computationally by aligning with respective reference strains with defined numbering. Sequence alignments to strains with known structure can also be used to build homology models and find the corresponding position of the mutation in the 3D structure. It turns out that D222/239G was located within the receptor binding pocket which determines the type of sugar-linked sialic acids recognized on human host cells but the precise effects on substrate specificity is still challenging to predict in detail by docking and modeling alone. Being able to switch between numbering schemes is also important to find prior work on related mutations in the literature. Indeed, a corresponding position in avian H1N1 has previously been investigated [44] as mutation G225D which is exactly equivalent to the new D222/225/239G but with inverted direction. The paper had found that G at this position is associated with preference for α2-3 avian-like receptor specificity while D would bind better to α2-6 human-like receptors. By analogy, it was possible to deduce that the new D222/225/239G mutation in the pandemic H1N1 could possibly shift the receptor preference to avian-like α2-3 receptors. The next important additional hint from the literature was that also humans have some α2-3 receptors but they are found deeper in the lungs, notably in the bronchiolae [45]. Finally, everything comes together and a hypothetical mechanism on how the new mutation could be related to severity is apparent where the D239G would change the receptor specificity to allow infections deeper in the lungs (Figure 1). More than a year later, this exact mechanism of the D222/225/239G mutation was studied in detail [46] and the experiments verified what could be suggested already much earlier by computational and literature analysis by a bioinformatics expert within a few hours. Many of the functions described here, have now been implemented in the WWW-based FluSurver that can accept patient-specific virus genome information and generate a clinical relevance report automatically (SMS *et al*., to be published).

There are many more examples where Bioinformatics analysis helped to elucidate phenotypic roles of new influenza mutations such as marker mutations of new variants rising in occurrence [47], changes in hemagglutinin surface epitopes [48] and glycosylation sites as well as detect known [49] and novel [50–52] mutations in the neuraminidase drug binding pocket that alter antiviral drug efficacy. While the wealth of prior work on influenza is crucial for the ability to make relevant computational predictions, it shows that, with a concerted effort, similar successes may be achieved in other areas of high interest.

### Conclusions from the sequence of the enterohemorrhagic O104:H4 *E. coli* strain

Next generation sequencing has dramatically brought down the cost of genome sequencing but the current reality is that there usually is a long way from the initial genomic data to information relevant for clinicians. However, there are exceptions. When an enterohemorrhagic O104:H4 *E. coli* strain caused a major outbreak in Germany [53] in 2011, the genome sequence was rapidly available through next generation sequencing [54]. At the same time, the Robert Koch Institute provided the microbial characterization including the clinically important antibiotic susceptibility profile [55]. In principle, the information if a specific antibiotic drug is effective against an organism should be encoded in its genome by the presence of the known target gene of the respective drug as well as the absence of associated drug resistance factors. Clearly, the prerequisite for computationally deriving an antibiotic susceptibility profile depends not only on the availability of the whole genome but also sufficiently complete annotation data for drug targets and resistance mechanisms of closely related strains or organisms. Since *E. coli* and related bacteria have been widely studied before in this regard, we show here that one can computationally identify antibiotic drugs that, potentially, can effectively target a new pathogen with available genome, such as the enterohemorrhagic O104:H4 *E. coli* strain. The steps to achieve this are essentially routine bioinformatics work but typically not easily accessible to clinicians.

First, the available genome sequences (http://www.ncbi.nlm.nih.gov/Traces/wgs/?val=AFOB01) were searched with BLASTX [56] for close to identical sequence matches against a database of known drug targets from DrugBank [57]. Requiring at least 97% sequence identity of the *E. coli* sequences to the proteins known to be drug targets ensures that also their structure will be highly similar and hence should represent the same drug binding properties. Second, we repeat the sequence search but this time against a database of known drug resistance factors from ARDB [58] requiring a lower threshold of at least 60% identity to conservatively pick up also more remote similarities to possible resistance factors. Third, we use a Perl script to parse the hits from the BLAST outputs as well as the drug target and resistance annotation data from the two databases and finally identify the list of drugs for which a known target gene was found in the genome but no respective associated resistance factor.

In order to validate the results, we compared our computational antibiotic susceptibility profile with the experimental results. To our positive surprise, 15 out of 25 experimentally tested antibiotics were also covered by the existing databases and could, hence, be assessed through our computational workflow. The identity thresholds for the two sequence searches described above have been selected to produce the best possible match with the experimental data. Table 1 shows that the *in silico* approach correctly assigns resistance or sensitivity for 13 of the 15 antibiotics. In detail, the new bacterial strain was correctly predicted to be sensitive to 7 antibiotics and resistant to 6 drugs from the list. The only two cases of a mismatch from the prediction with the clinical experimental result are interesting and discussed below.

**Table 1:** **Predicted potentially effective drugs against enterohemorrhagic** ***E. coli***

Antibiotic	Exp.	Comp.
Piperacillin/Tazobactam	R*	S
Cefoxitin	R	**R**
Ceftazidim	R	**R**
Cefpodoxim	R	**R**
Imipenem	S	**S**
Meropenem	S	**S**
Amikacin	S	**S**
Gentamicin	S	**S**
Kanamycin	S	**S**
Tobramycin	S	**S**
Streptomycin	R	**R**
Tetracyclin	R	**R**
Nitrofurantoin	S	**S**
Trimethoprim/Sulfamethoxazol	R	**R**
Fosfomycin	S	**R**

Experimentally measured (Exp.) versus computationally predicted (Comp.) antibiotics susceptibility profile. R … resistant; S… sensitive; * … defined as resistant (AES VITEK). Prediction and experimentally determined results coincide except for two cases (Piperacillin/Tazobactam and Fosfomycin) which are discussed in the text in detail.

The first case is the combination drug Piperacillin/Tazobactam which we flag as sensitive but the Robert Koch Institute as resistant. Sequence searches identified a TEM-1 metallo beta-lactamase in O104:H4 *E. coli* which causes resistance to penicillins (including Piperacillin) by degrading them but we also find that there exists a specific inhibitor against TEM-1 metallo beta-lactamases, Tazobactam, which is given in combination with Piperacillin to inhibit the beta-lactamase and, therefore, increase efficacy of penicillins to which this strain should otherwise be resistant. In theory, this means that the computational prediction that Piperacillin/Tazobactam is effective should be correct. However, it turns out that, in clinical practice, this drug is recommended to be avoided due to possible inoculum effects. Hence, the resistant flag from the clinical judgement according to the used VITEK AES experimental classification system.

The second case is Fosfomycin, to which the new strain was experimentally found to be sensitive while the computational approach assumed resistance due to the identification of a multidrugefflux pump protein annotated to also export Fosfomycin. This means that either the annotation is inaccurate or it would be interesting to further look into the detail of the few sequence differences between the new and the previously known transporter (99% identity) to find determinants of activity and substrate specificity which could be considered in a future more comprehensive approach.

Overall, this crude workflow utilizing available databases shows that a computational antibiotics susceptibility profile can be derived with some accuracy by combining next generation genome sequencing with further computational analysis, but it definitely still needs a critical experienced doctor who further scrutinizes and selects the most suitable treatment according to the circumstances of the infected patient as well as includes any new clinical findings on drug responses of the respective strain.

### Bacterial communication and cooperation in health and disease

The analysis of human microbiomes and small bacterial communities causing multi-bacterial diseases are among the most challenging and intriguing tasks of medical genome research today [59–61] also including the field of plant diseases [62]. The discovery of chemical communication among bacteria in the 1990s has fundamentally changed the traditional view that pictures bacteria as single-celled organisms living in isolation [63–66]. In the last fifteen years, it has become increasingly evident that bacteria have the potential to establish highly complex communities. Many microbes live in large, multispecies communities in which the participants jointly exploit the resources. Multispecies microbial consortia constitute a major form of life that is found in environments ranging from high-altitude mountains (more than 8 km above sea level) to more than 10 km below the surface of the oceans, and have always been among the most important members and maintainers of the planet's ecosystem. The medical importance of this phenomenon is sweeping. Opportunistic pathogenes, such as *Pseudonomas* and *Burkholderia* species abound in hospital environments, ready to attack patients weakened by disease or injury. For instance, *Pseudomonas aeruginosa* usually does not harm a healthy human organism, but can be lethal in the lung of cystic fibrosis (CF) patients, or in burn wounds [67].

Many prokaryotes possess inter-cellular signaling systems which allow species to colonise new habitats, to invade hosts and to spread over surfaces [63–66]. A typical example is quorum sensing (QS) which enables bacteria to switch from low activity to high activity regimes using signaling molecules as well as „public goods” (e.g. surfactants, enzymes, siderophores) that facilitate movement, nutrient uptake amongst other things [65, 66]. We share the widespread opinion that the „change of bacterial lifestyle” is crucial for colonizing habitats and infecting susceptible hosts – unfortunately the signalling systems that orchestrate the underlying communication and collaboration mechanisms are not accurately annotated in bacterial genomes. Therefore, a systematic characterization of QS systems in Gram negative bacteria was carried out [68, 69] and a modelling effort to map out the theoretically possible consequences of communication and collaboration in bacterial populations was initiated [70–72]. Virulence and adaptability of many Gram-negative bacterial species are associated with an N-acylhomoserine lactone (AHL) gene regulation mechanism called quorum sensing (QS). The arrangement of quorum sensing genes is variable throughout bacterial genomes, although there are unifying themes that are common among the various topological arrangements. A bioinformatics survey of 1403 complete bacterial genomes revealed characteristic gene topologies in 152 genomes that could be classified into 16 topological groups [68, 69]. A concise notation for the patterns was developed and it was shown that the sequences of *LuxR* regulators and *LuxI* autoinducer synthase proteins cluster according to the topological patterns.

The macroscopic behavior of bacterial communities is notoriously difficult to study, colony patterns, invasion/colonization events depend on a multitude of parameters many of which cannot be reproduced in lab cultures. Therefore, computational modeling, and particularly the use of simplified minimal models is a very important tool for studying the behavior of populations in rational terms. Agent-based models of communicating and collaborating bacteria have developed [70]. The bacterial cells are represented by agents randomly moving on a plain (such as an agar surface), while consuming nutrients, secreting signal molecules and “public goods”. Nutrients, signals and public goods are diffusing on the surface, and their local concentration exceeds a threshold, the metabolism and movement of bacterial agent switches to a more intensive state. In this model signals are the means of communications, and public goods are the means of cooperation as can be observed in QS bacteria. Even though highly simplified, the model reflects the crucial behavior patterns of communicating/cooperating bacteria in an open, nutrient/limited environment. Namely, 1) isolated bacteria cannot survive; only bacteria reaching a critical population size (“quorum”) have a chance for survival. 2) Bacteria self-organize into compact communities or “active zones” in which signals and public goods are present in sufficient amounts [70]. 3) Collaborating communities can collapse if non-cooperating mutants are present [71, 72].

Modeling the mutants of QS mechanisms is highly relevant for disease prevention. There is a very vivid interest from the pharmaceutical and pesticide industries, analysts agree that interventions targeting quorum sensing are among the major trends of the future. Since many bacteria use quorum sensing for infection, it is plausible to think about jamming strategies. According to one such scenario, one can saturate the surface of a plant with a signal molecule that will call bacteria to attack. If a lonely pathogen lands on the surface, it will immediately start to attack, but at the wrong time and place. Since it is alone, it will perish. Or, we can put a gene into the plant that produces an enzyme capable of destroying the signal molecule of the pathogenic bacteria, so that those will never wage an attack. But both strategies can strike back since they can also destroy the signaling of the beneficial bacteria that are essential to the host. According to a third scenario one may prevent the growth of an infecting pathogen by a greedy but antibiotic sensitive mutant of the same species, and then we eliminate the mutant by an antibiotic that specifically acts on that mutant. This is very appealing, but what do we do if the mutant created to heal gets some harmful genes or looses its antibiotic susceptability? Many similar questions can be studied using computational models [73].

## Impact of bioimage informatics on healthcare

Most likely, the penetration of automated evaluation tools for the analysis of clinically relevant histological images in diagnostic contexts is one of the areas that will experience great changes in the near future. The process of biomedical imaging involves little or no discomfort to the patients, while providing an effective tool for diagnosis. However, successful usage of images requires a high level of human intelligence, making automated image analysis by machines a challenging task. Currently, the gold standard for diagnosis through imaging is by experienced clinicians, typically radiologists or pathologists. It takes many years to train proficient clinicians to analyze images manually and, despite that, this gold standard is not perfect and suffers from subjective variations between different clinicians.

Advances in image processing, pattern recognition and computer vision in the past decades have boosted the possibilities for the application of computing technology. Currently, the focus is on computer aided diagnosis rather than to achieve a fully automated approach. Software that can support decision making and reduce the workload of clinicians, especially in routine operations, is extremely useful and valuable. Besides the direct derivation of clinically relevant conclusions from the images, such systems call also for the integration with databases of medical ontologies, the patients’ medical records, etc.

Computational image analysis methods can be broadly categorized into those used for assessment, diagnosis and surgery. This section attempts to cover several exemplary areas of imaging and image analysis in healthcare. Because of the large extent of research work ongoing in academic bioimage informatics and medical image analysis and the growing engagement of the industry, this section cannot be comprehensive but rather we seek to cover a broad spectrum.

### Digital pathology

Advances in computer vision and microscopy instrumentation have made digital pathology an important emerging field. The objective is to aid the pathologist in the analysis of high resolution cellular images obtained through biopsy. For example, highlighting regions of interest or reducing diagnostic variation can generate a big impact. Histological images from various organs such as prostate [74], breast [75] and liver have been the object of algorithm development.

Here, we shall focus our discussion on prostate digital pathology. Prostate cancer has a high prevalence rate worldwide. For example, it is the most common non-cutaneous male cancer in the United States [76] and it is the 3rd most common male cancer in Singapore [16]. The American Cancer Society report in 2009 estimates 192,280 new prostate cancer cases with 27,360 prostate cancer specific death [76]. The severity of prostate cancer diagnostics is compounded by disagreements between individual pathologists with regard to grading using the Gleason classification [77]. This agreement between different pathologist can be as low as 70% [78] and up to 29% of Gleason gradings were different between pre- and post-operative prostate cancer specimen [79]. Hence, having objective computer algorithms to aid in prostate pathology assessment is essential to improve diagnosis.

Most computational methods are developed to analyze microscopy images on the standard hematoxylin/eosin stain. The goals are gland segmentation since the architecture of glands is critical for Gleason grading and the identification and segmentation of nuclei since this is useful for detecting nuclei signatures specific to cancerous cells. Common computer vision techniques used are level sets [80], fractal analysis [81] and machine learning [80, 82–86]. These techniques are used to segment glands [80, 85] and nuclei [82, 84] or to identify regions of malignancy directly [83].

### Computer vision in dermatology

Assessment of skin condition and health is both important for clinical medicine as well as for the cosmetics industry. At present, assessment of the skin typically involves a trained dermatologist who will examine features such as textures and landmarks. While training of dermatologists takes many years, the subsequent diagnosis suffers from subjective interpretation differing among dermatologists. Hence, a more objective approach is in demand.

Considerable effort is ongoing to analyze skin surfaces through the use of objective computational methods. Protocols to ensure objective and consistent imaging of human skin (for example, in a well-controlled lighting environment) are vital for reliable diagnosis by computer algorithms [87–89]. Image acquisition is followed by the application of task-dependent image processing and computer vision methods. Liu *et al*. [90] use texture analysis to create an objective way of evaluating the effectiveness of treatment. A neural network framework has been developed to analyze the human skin conditions such as color, roughness, glossiness or tension [91, 92]. Skin images have also been studied with data mining methods [88, 93] and via modeling/reconstructing the skin surface [89, 94].

### Computer vision in eye diseases

Imaging methods for eye diseases are unique among bioimaging techniques because images of the eyes are easily accessible using conventional light cameras. There is no need for expensive and sophisticated machines such as a computer tomograph or magnet resonance imager. A common imaging modality is the optical coherence tomography; other imaging methods such as fundus photography, ultrasound and infra-red imaging are also used. Although image analysis has been used in the assessment of many eye diseases, we will focus our discussion on glaucoma and dry eye disease in this paper.

#### Angle closure glaucoma

According to a world health organization report [95], glaucoma is a major global cause of blindness (approximately 5.2 million cases and about 15% of all cases of blindness). The impact of glaucoma on public health will increase with an aging population. However, the lack of a comprehensive measure of glaucoma compounded with its ability to cause sudden blindness makes it hard for treatment planning. Surprisingly, about 50-90% of potential patients in the world are unaware that they have glaucoma [96, 97].

Glaucoma is classified into angle closure and open angle glaucoma according to the drainage angle, the angle between the cornea and iris. Primary angle closure glaucoma is the major form of glaucoma in Asia, in particular, among the Chinese population. It was suggested that angle closure glaucoma causes more blindness than open angle glaucoma in relative terms [98].

A common way for assessment of angle closure glaucoma is through gonioscopy in which the doctor uses an optical instrument to look at the anterior chamber to decide if the drainage angle is open or close. Ultrasound [99] and optical coherence tomography (OCT) [100] images are also used for assessment. Computer vision techniques are used for analyzing eye images derived from the different modalities. As it takes much effort to master the technique of gonioscopy, Cheng *et al*. [101] developed a computational technique for RetCam images. A machine-learning based method aids glaucoma diagnosis by analyzing the cup-to-disc ratio measured on fundus images [102]. OCT images provide high resolution and a 3D view of the anterior chamber. Image analysis software has been developed to make precise measurements of important geometric information such as anterior chamber area, anterior chamber width, iris thickness, etc. on OCT images [103]. These data can then be correlated to generate new clinical knowledge [104, 105].

#### Image analysis in assessing the dry eye condition

The disease of dry eye has no clear definition; generally, it is a condition in which there is an unstable tear film during the open eye state. The dry eye condition has a prevalence rate of 10-20% in Sweden, Japan, Australia and several other countries. The most common treatment of dry eye is application of eye drops [106].

One cause of dry eye disease is meibomian gland dysfunction. The meibomian glad is located at the inside of the tarsel plate that supplies meibum, an oily substance, which forms a protective layer to the tear film. Dysfunction of meibomian glands causes lack of meibum and, often, resulted in degeneration of meibomian glands.

The morphology of meibomian glands can be imaged using an infra-red camera mounted on a conventional slit lamp camera [106]. This imaging technology has enabled the application of advanced computer vision techniques for better diagnosis and patient management. Images from healthy meibomian glands shows a strip like pattern in gland morphology; with the strips being relatively straight, parallel and equally spaced. Images of highly degenerated glands show no strip like patterns at all, but only small isolated regions of remnant glands. Morphology for early stage disease shows twisting, non-parallel and unequally spaced strip like patterns [106].

While the process of imaging is simple and relatively cheap, the analysis of the morphology of meibomian glands and other clinical examinations that eventually lead to diagnosis and treatment require trained ophthalmologists with experience in handling dry eye patients. Unfortunately, there is no clear objective criteria for grading meibomian glands morphology degeneration, although some schemes have been suggested [106]. Inter-individual variation will also cause problems. Hence, large population screens on meibomian glands morphology does not directly lead to overall increase in better management of the disease.

An effective way to circumvent the problem of cost and inter-individual variation is to develop advanced computer vision techniques to process and grade images of meibomian glands. A team from Singapore has developed an image analysis software that can enhance infra-red images of meibomian glands, segment the strip-like patterns and extract important features for classifying the images [107].

### Image analysis for assisted surgery

Pre-planning is an important component to the success of surgery, so that surgical operations can be performed systematically, completely and swiftly. Usually, planning involves studies of 3D images of the part of the patient’s body where the operation will be performed. Image assisted surgery is available or being developed for almost all parts of the human body, for example for brain, liver, heart, gastrointestinal tract and for hand reconstruction surgery. The digital 3D image is enhanced by advanced computer graphics, visualization and various forms of accurate geometrical measurements done by the computer. This enhancement is very important because the human mind cannot decipher 3D objects represented on a 2D computer screen effectively. We are also unable to make accurate geometrical measurements. In this case, the computer essentially provides the “ruler” to make measurements.

#### Tumor segmentation

Accurate measurements are particularly important in the case of surgery aimed at removing tumors. The size of the tumor is an important prognostic factor for treatment. 1D and 2D measurements such as tumor length, the largest axis length or cross sectional area had been used as a measure of tumor sizes. However, studies have shown that tumor volume provides a more accurate estimate of the tumor size [108, 109]. Accurate measurement of tumor sizes calls for effective segmentation of tumors. Once properly segmented, the tumor size can be calculated trivially. Tumors occur in many parts of the human body and different segmentation algorithms are developed for segmenting tumors in different organs. The literature in this area is vast. In the following, we focus on liver tumors. Liver cancer accounts for about one million deaths per year [110]. Segmentation is usually done on computer tomography images. Many techniques have been developed to segment liver and its tumor including region growing [109, 111], statistical techniques [109], machine learning [108, 109], active contours [112], fuzzy c-means [113] and watershed [114].

Surgery planning also needs careful consideration of the vasculature structure around the tumor and their relationship with the tumor. Hence, segmentation of the vasculature structure can aid the surgeon to visualize the structure and location of vessels [115].

## Concluding remarks

The development and implementation of analytical and computational tools provided from the side of bioinformatics and bioimaging analysis provide opportunities for quality interaction among biotechnology, fundamental life science research and clinical studies. Bioinformatics findings can be translated into innovations that are adopted by the healthcare system and biomedical industry in form of diagnostic kits, analysis programs, etc. after the validation in both bench and clinical studies. In this article, we present several examples of how clinically relevant conclusions can be drawn from sequencing, expression profiling or histopathological bioimaging data with computational biology algorithms.

Unfortunately, considerable basic research is still necessary to make full use of the potential opportunities that are associated with the increasing availability of high-throughput technologies such as genome sequencing, mainly since most of the genome’s hidden functional information is not known; the understanding of biomolecular mechanisms that translate genotype into phenotype is limited. But the progress in this field is uneven; pathogen sequencing can already provide important insights in contrast, for example, to sequencing of cancer samples.

Since an efficient healthcare system must be aligned to social, economic and political infrastructure of the country and focus on evidence-based prophylactic, prevention, diagnosis, prognosis, prediction and treatments that are proven to provide quality service and clinical outcome in a cost-effective manner, genomics, proteomics and other new technologies will first have to demonstrate in a research hospital setting that they can have a dramatic effect in improving health care, also cost-wise in addition to providing better quality of life, before the approaches will penetrate the routine healthcare systems. Nevertheless, it is very clear that major advances in diagnostics and treatments for infections as well as cancers, circulatory and metabolic diseases that are critical for improving most healthcare systems will arise from these developments in a medium to longer time frame.

As we have seen above, genome information of pathogens linked with the geographic origin allows tracing the spread of infections and parasites. Similarly, analyzing the geographic, even better spatio-temporal distribution of disease occurrences can provide hints for environmental influences [116, 117]. Generally, going beyond the patient-centric approach and the linking of biomolecular and clinical data of populations with geographic information, data on food and environment, etc. will be an important source for improving public health, for stopping epidemics, for finding sources of food or environmental poisoning and for improving life styles.

## Authors’ information

VK, HKL, SMS and BE are principal investigators at the Bioinformatics Institute (BII) of the Agency for Science, Technology and Research in Singapore; FE is the director of this institute. MJM is the director of the Institute of Genomic Medicine and Rare Disorders at Semmelweis University in Budapest. SP is a senior scientist and group leader of the Protein Structure and Bioinformatics Laboratory at the ICGEB in Trieste and professor at the Pázmány Péter Catholic University in Budapest.
